# Late relapse of ulcerative colitis presenting as tracheobronchitis: a case report

**DOI:** 10.1186/s13256-022-03583-5

**Published:** 2022-11-02

**Authors:** Shouichi Okamoto, Kengo Koike, Mitsuaki Sekiya, Koichi Nishino, Tomoyasu Mimori, Kazuhisa Takahashi

**Affiliations:** 1 Division of Respiratory Medicine, Juntendo University Faculty of Medicine and Graduate School of Medicine, 2-1-1 Hongo, Bunkyo-ku, Tokyo, 113-8421 Japan; 2 Department of Respiratory Medicine, Saiseikai Kawaguchi General Hospital, 5-11-5 Nishikawaguchi, Kawaguchi, Saitama 332-8558 Japan

**Keywords:** Bronchoscopy, Case report, Inflammatory bowel disease, Leucine-rich α-2-glycoprotein, Ulcerative colitis, Tracheobronchitis

## Abstract

**Background:**

Lung involvement in inflammatory bowel diseases usually follows colitis. However, the time to lung involvement onset varies depending on the case, and pulmonary lesions are usually not parallel to exacerbations of the colitis.

**Case presentation:**

A 67-year-old Asian woman with a 38-year history of ulcerative colitis presented to our hospital with a complaint of prolonged dry cough for 2 months. The colitis had remained quiescent for > 35 years with low-dose salazosulfapyridine treatment. Chest computed tomography indicated circumferential thickening of the tracheal wall, while bronchoscopy examination revealed widespread erythematous edema and diffuse narrowing of the bronchial lumen. Biopsy of the bronchial mucosa showed submucosal lymphocytic infiltration. She was diagnosed with ulcerative-colitis-related tracheobronchitis and successfully treated with corticosteroids.

**Conclusions:**

Tracheobronchitis, in our case, occurred despite the longest remission period previously reported. Careful follow-up is necessary for the early recognition and treatment of pulmonary disease in patients with ulcerative colitis, regardless of the disease duration and long-term remission of colitis.

## Background

Ulcerative colitis (UC), which usually develops during young adulthood, is a chronic inflammatory and immunologically mediated disease that mainly affects the colon. It is characterized by remission and relapses [1]. Since the first report of ulcerous bronchitis associated with UC [2], the cumulative literature has revealed that respiratory manifestations of inflammatory bowel diseases (IBDs), namely UC and Crohn’s disease (CD), are more common and exist in various forms other than those generally recognized [3]. This has been possible through the popular use of high-resolution computed tomography (HRCT).

IBD onset usually precedes pulmonary symptoms [4, 5], and previous studies have reported that colorectal resection might trigger a shift in the inflammatory process from the resected bowel to the lungs and be involved in the pathogenesis of pulmonary manifestations in patients with IBD [5, 6]. Crosstalk between bowel disease and airway disorders has been suggested. However, to date, neither the exact mechanism nor the onset of crosstalk has been elucidated [7].

Herein, we describe a rare case of UC with diffuse tracheal and bronchial involvement. Our patient suffered from tracheobronchitis and had, to the best of our knowledge, the longest remission period of colitis previously reported.

## Case presentation

A 67-year-old Asian woman, who was a nonsmoker, presented to our hospital with a complaint of prolonged dry cough for 2 months. The patient also had dyspnea in the supine position, night sweats, and mild appetite loss for 1 month, but no complaints of any gastrointestinal symptoms. She was diagnosed with UC at the age of 29 years with bloody diarrhea, had been in long-term remission for over 35 years, and was taking salazosulfapyridine (1000–1500 mg/day) orally. She had no history of occupational dust exposure, but had a family history of pulmonary tuberculosis.

On physical examination, her vital signs were as follows: temperature, 36.4 °C; heart rate, 99 beats per minute; blood pressure, 136/74 mmHg; respiratory rate, 16 breaths per minute; and oxygen saturation on room air, 98%. Auscultation of the neck and chest revealed normal breath sounds, without adventitious sounds. The rest of the physical examination, including the outer ear, nose, and joints, was normal.

Blood analysis revealed elevated C-reactive protein (CRP; 10.71 mg/dL) and increased levels of leucine-rich α-2-glycoprotein (LRG; 44.2 μg/mL, normal < 16.0 μg/mL), a new biomarker for evaluating disease activity in patients with UC [8]. Tumor markers, proteinase-3 anti-neutrophil cytoplasmic antibody, soluble interleukin (IL)-2 receptor, and angiotensin-converting enzyme were within normal limits, and T-SPOT.TB (an interferon-γ release assay) was negative.

**Fig. 1:**
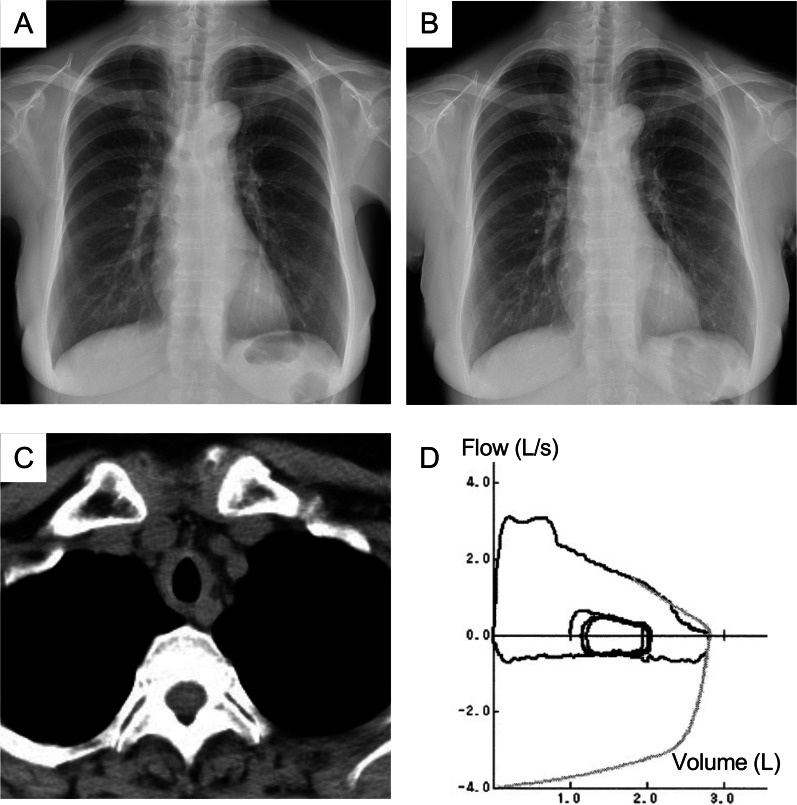
Chest X-rays obtained at the first visit (**A**) and 1 year before the first visit (**B**), respectively. Chest X-ray at the first visit (**A**) showing narrowing of the trachea compared with that from a year earlier (**B**). **C** Chest computed tomography image showing circumferential thickening of the tracheal wall. **D** Flow–volume loop graph demonstrating fixed upper airway obstruction

Chest radiography revealed narrowing of the trachea (Fig. 1A) compared with the previous year (Fig. 1B). Chest computed tomography (CT) demonstrated circumferential thickening of the tracheal wall, ranging from the trachea to the bilateral main bronchi (Fig. 1C), without lung parenchymal abnormalities. Pulmonary function tests showed a forced vital capacity of 2.81 L [114.5% of the predicted volume (pred)], forced expiratory volume in 1 second of 2.06 L (101.5% pred), and decreased peak expiratory flow of 3.11 L per second (53.3% pred). A flow–volume loop graph at the first visit revealed fixed upper airway obstruction (Fig. 1D).

**Fig. 2:**
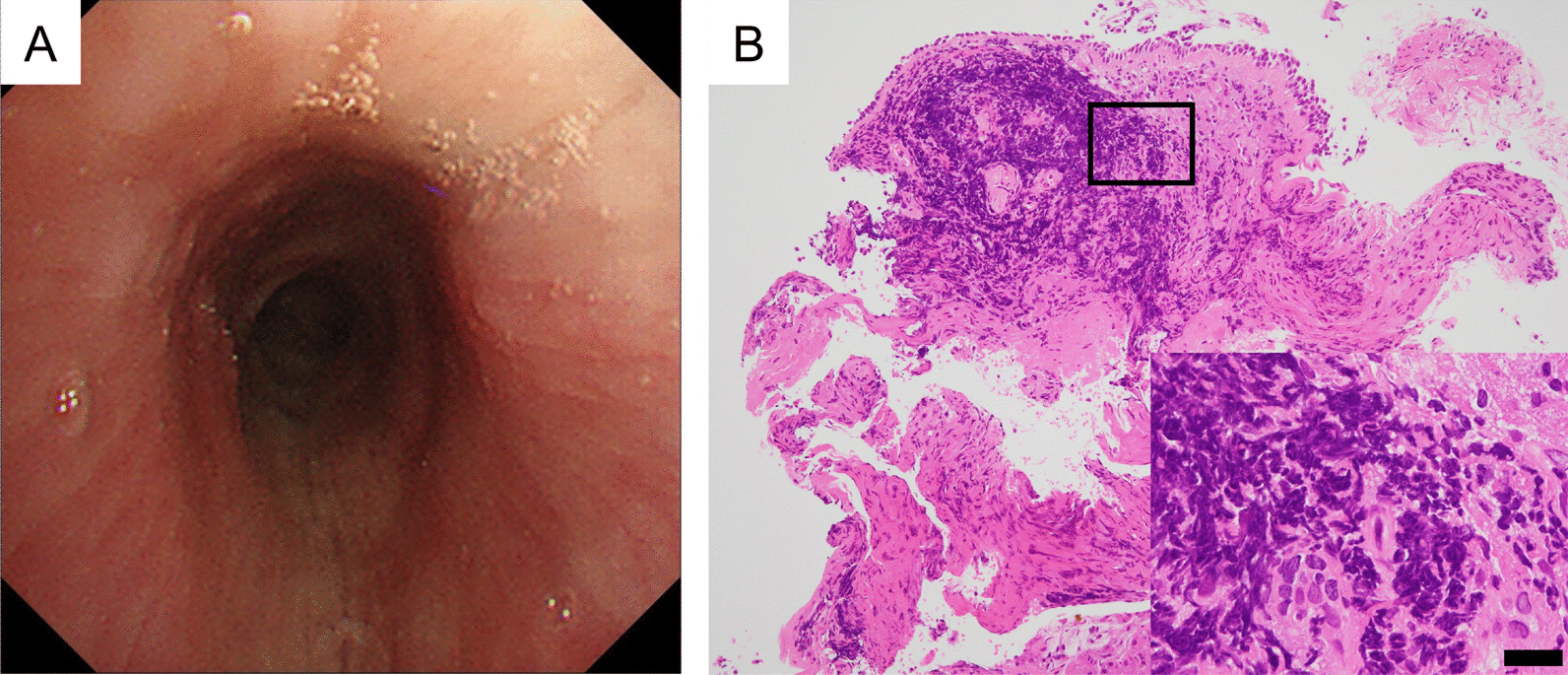
**A** Bronchoscopic examination showing diffuse narrowing of the trachea with inflamed erythematous mucosa. **B** Photomicrograph of endotracheal mucosal biopsy specimen showing intense submucosal infiltration. The inset within **B** is a magnified view of lymphocytic infiltration. Scale bar, 20 μm

Her symptoms were unresponsive to treatment with oral levofloxacin (500 mg/day) or inhaled fluticasone furoate (100 μg/day)/vilanterol trifenatate (25 μg/day) administered for 1 week. Bronchoscopy performed to investigate extensive large airway disease revealed widespread edema with erythema and diffuse concentric narrowing of the bronchial lumen from the trachea to the bilateral segmental bronchi (Fig. 2A); however, neither swelling nor paralysis of the vocal cord could be confirmed. Dynamic collapse of the central airway was not observed during respiration. Mucosal biopsy specimens revealed marked submucosal lymphocytic infiltration (Fig. 2B). No evidence of infection, malignancy, vasculitis, or amyloidosis was found. After thorough examination, the patient was diagnosed with UC-related tracheobronchitis and was administered oral prednisolone at a starting dose of 40 mg/day.

**Fig. 3:**
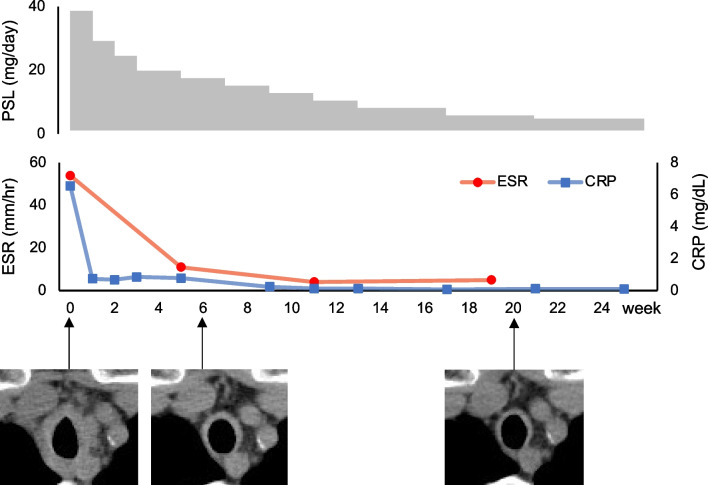
Clinical course from initiation of corticosteroid therapy. Chest computed tomography images obtained at the first visit, 1.5-month follow-up, and 5-month follow-up show amelioration of bronchial wall thickening. *CRP* C-reactive protein, *ESR* erythrocyte sedimentation rate, *PSL* prednisolone

The patient’s symptoms improved expeditiously, and the prednisolone dose was gradually reduced. Finally, dyspnea completely resolved at a dose of 25 mg/day. Repeat chest CT performed at 1.5- and 5-month follow-up showed improvement in bronchial wall thickening (Fig. 3). CRP and erythrocyte sedimentation rate (normal < 16 mm/hour in females) decreased and remained within the normal range with oral prednisolone dose of 4 mg/day (Fig. 3). Similarly, LRG decreased to a near-normal level (17.6 μg/mL) at 6-month follow-up.

## Discussion and conclusions

Although IBDs are associated with several comorbidities that occur at extraintestinal sites, pulmonary involvement is relatively rare. The respiratory system is derived from the primitive gut tube, a precursor of the gastrointestinal tract. Both the lungs and the gastrointestinal tract contain submucosal lymphoid tissue that contributes to host immune defense. Bowel and lung involvement, indicating a shift in the inflammatory process, at times occurs soon after colorectal resection [5, 6]. Therefore, basic research is needed to elucidate crosstalk between the respiratory and gastrointestinal immune systems in patients with IBD. The underlying mechanism is yet to be understood; however, it is speculated that dysfunctional immune-cell homing, dysbiosis-driven immune response, and systemic inflammation may be involved in pulmonary manifestations in patients with IBD [7].

In most cases of IBD, pulmonary involvement follows bowel disease [4, 5]. The time to onset of lung involvement differs in each case (range 1–25 years) [4]. In addition, pulmonary lesions are not parallel to IBD exacerbations and may occur during remission [9]. Three cases of tracheobronchitis that occurred over 30 years from the onset of UC have been reported [10–12]. These cases developed during remission, and the durations between pulmonary involvement and start of remission were 15, 13, and 29 years, respectively [10–12]. All patients were occasional smokers or nonsmokers. A meta-analysis showed that, while current smoking is protective against UC development, smoking status is associated with CD development [13]. To the best of our knowledge, our patient developed tracheobronchitis despite having the longest remission period previously reported. Observations from previous and present cases imply that the possibility of relapse as a pulmonary disease should always be considered, especially in nonsmokers.

The prevalence of thoracic abnormalities on HRCT ranges from 22% to 89% in patients with IBD [14, 15]. The sites of involvement include the upper airways and the large and small airways of the lungs, and concomitant diseases may also be present. Large airways, comprising the bronchi from the level of the lobar bronchi to the level of terminal bronchioles, are the most common anatomical locations of respiratory manifestations in IBDs, accounting for approximately 50% of all cases [14]. Upper airway disease (UAD) is responsible for 8% of all pulmonary manifestations in IBDs and includes tracheobronchitis, laryngeal, and glottic edema or stenosis [3, 14]. UAD is usually associated with cough, hoarseness, and dyspnea, and physical examination may reveal wheezing or stridor. It has been documented that UC-related tracheobronchitis shows an obstructive spirometry pattern [10].

Occasionally, chest radiography may aid in the detection of tracheal narrowing. Chest CT can reveal circumferential or nodular thickening of the tracheobronchial wall. The differential diagnoses of UAD, especially tracheobronchial wall thickening, associated with IBDs, include bronchial tumors (benign or malignant tumors or lymphoma), relapsing polychondritis, granulomatosis with polyangiitis, sarcoidosis, and amyloidosis. Bronchoscopic findings of tracheobronchitis in IBDs comprise widespread mucosal edema, narrowing of the bronchus, occasional ulceration of the mucosa, multiple nodules, and bronchial stenosis [10]. Bronchial biopsy typically demonstrates lymphoplasmacytic or lymphocytic inflammation of the submucosa with no evidence of malignancy, vasculitis, amyloidosis, or infection [10]. In previous cases, supporting findings of IBD-associated pulmonary involvement were nonspecific. Therefore, as in our case, the diagnosis of IBD-associated UAD was made on the basis of the patient’s history, CT and bronchoscopic findings, and pathological findings in biopsy after excluding other possible causes.

Patients with UC with mild-to-moderate colitis who do not respond to or achieve remission with 5-aminosalicylic acid drugs have been treated with corticosteroids. In contrast, corticosteroids have mainly been used in patients with IBD with comorbid pulmonary diseases, despite a lack of clinical trial data [3]. However, 12.5% of such patients did not respond or showed only a marginal improvement following corticosteroids [16]. Azathioprine has been used as a steroid-sparing or additional treatment agent in patients with UC and UAD [10, 17]. Additionally, in one case, infliximab, an anti-tumor necrosis factor-alpha (TNF-α) monoclonal antibody, was effective against UC-related tracheobronchitis refractory when combined therapy with high-dose inhaled or systemic corticosteroids and azathioprine was administered [18]. Further research is warranted to validate the optimal combination therapy in patients with UC with pulmonary manifestations. The present case showed rapid improvement with oral corticosteroids and maintained remission with prednisolone at 4 mg/day; however, the bronchial wall thickening had not subsided completely. Therefore, careful monitoring is necessary to identify relapse during weaning or cessation of corticosteroids.

CRP and serum amyloid A (SAA) have been conventionally used as biomarkers of disease activity in patients with UC because proinflammatory cytokines such as interleukin (IL)-6, IL-1β, IL-22, TNF-α, and interferons are upregulated in IBDs, and IL-6 plays a central role in the induction of CRP and SAA [19]. However, they often remain at normal levels despite active inflammation of the colon [20]. LRG is a 50-kDa glycoprotein produced by neutrophils, macrophages, hepatocytes, and intestinal epithelial cells [8]. LRG expression is stimulated not only by IL-6 but also by TNF-α and IL-22 [8]. Serum LRG concentrations were found to be significantly elevated in patients with UC with active bowel inflammation compared with those in remission and correlated with disease activity in UC better than CRP [8]. In our case, the decrease in LRG levels was consistent with disease activity at the 6-month follow-up after corticosteroid treatment. Therefore, the serum LRG level might be a useful biomarker of disease activity and may be used to monitor patients with UC for pulmonary diseases.

In conclusion, we present a case of UC-related tracheobronchitis that occurred 38 years after the onset of UC. Clinicians should consider the possibility of late-onset pulmonary disease in patients with UC regardless of the disease and long-term remission.

## Data Availability

All data generated or analyzed during this study are included in this published article.

## Abbreviations

CD: Crohn’s disease
CRP: C-reactive protein
CT: Computed tomography
HRCT: High-resolution computed tomography
IBD: Inflammatory bowel disease
IL: Interleukin
LRG: Leucine-rich α-2-glycoprotein
SAA: Serum amyloid A
TNF-α: Anti-tumor necrosis factor-alpha
UAD: Upper airway disease
UC: Ulcerative colitis
